# Trends in percentages of gestational diabetes mellitus attributable to overweight, obesity, and morbid obesity in regional Victoria: an eight-year population-based panel study

**DOI:** 10.1186/s12884-022-04420-9

**Published:** 2022-02-01

**Authors:** George Mnatzaganian, Mark Woodward, H. David McIntyre, Liangkun Ma, Nicola Yuen, Fan He, Helen Nightingale, Tingting Xu, Rachel R. Huxley

**Affiliations:** 1 Rural Department of Community Health, La Trobe Rural Health School, La Trobe University, Bendigo, Victoria Australia; 2 The Peter Doherty Institute for Infection and Immunity, Melbourne, Victoria Australia; 3 The George Institute for Global Health, Imperial College, London, UK; 4 The George Institute for Global Health, University of New South Wales, Sydney, Australia; 5 Obstetric Medicine, Mater Health Services, University of Queensland, Brisbane, Queensland Australia; 6 Department of Obstetrics and Gynecology, Peking Union Medical College Hospital, Beijing, China; 7 Department of Women’s & Children’s Services, Bendigo Health, Bendigo, Victoria Australia; 8 John Richards Centre for Rural Ageing Research, La Trobe Rural Health School, La Trobe University, Albury-Wodonga, Victoria Australia; 9 Rural Department of Nursing and Midwifery, La Trobe Rural Health School, La Trobe University, Bendigo, Victoria Australia; 10 Department of Health Policy and Management, School of Public Health, Capital Medical University, Beijing, China; 11 Faculty of Health, Deakin University, Melbourne, Australia

**Keywords:** Gestational diabetes mellitus, Obesity, Population attributable fractions, Incidence, Trends

## Abstract

**Background:**

Gestational diabetes mellitus (GDM) is the fastest growing type of diabetes in Australia with rates trebling over the past decades partially explained by rising obesity rates and maternal age among childbearing women. Percentage of GDM attributable to obesity has been documented, mostly focusing on metropolitan populations. In parts of regional (areas outside capital cities) and rural Australia where overweight, obesity and morbid obesity are more prevalent, intertwined with socioeconomic disadvantage and higher migrant communities, trends over time in adjusted percentages of GDM attributed to obesity are unknown.

**Methods:**

In this population-based retrospective panel study, women, without pre-existing diabetes, delivering singletons between 2010 and 2017 in a tertiary regional hospital that serves 26% of Victoria’s 6.5 million Australian population were eligible for inclusion. Secular trends in GDM by body mass index (BMI) and age were evaluated. The percentage of GDM that would have been prevented each year with the elimination of overweight or obesity was estimated using risk-adjusted regression-based population attributable fractions (AFp). Trends in the AFp over time were tested using the augmented Dickey-Fuller test.

**Results:**

Overall 7348 women, contributing to 10,028 births were included. The age of expecting mothers, their BMI, proportion of women born overseas, and GDM incidence significantly rose over time with GDM rising from 3.5% in 2010 to 13.7% in 2017, *p* <  0.001, increasing in all BMI categories. The incidence was consistently highest among women with obesity (13.8%) and morbid obesity (21.6%). However, the highest relative increase was among women with BMI < 25 kg/m^2^, rising from 1.4% in 2010 to 7.0% in 2017. Adjusting for age, country of birth, socioeconomic status, comorbidities, antenatal and intrapartum factors, an estimated 8.6% (confidence interval (CI) 6.1–11.0%), 15.6% (95% CI 12.2–19.0%), and 19.5% (95% CI 15.3–23.6%) of GDM would have been prevented by eliminating maternal overweight, obesity, and morbid obesity, respectively. However, despite the rise in obesity over time, percentages of GDM attributable to overweight, obesity, and morbid obesity significantly dropped over time. Scenario analyses supported these findings.

**Conclusions:**

Besides increasing prevalence of obesity over time, this study suggests that GDM risk factors, other than obesity, are also increasing over time.

**Supplementary Information:**

The online version contains supplementary material available at 10.1186/s12884-022-04420-9.

## Background

In 2013, the International Diabetes Federation (IDF) estimated that 16.8% of live births were to women with some degree of hyperglycaemia in pregnancy [1], with the vast majority being attributable to gestational diabetes mellitus (GDM) [2]. The economic burden of GDM extends to the diagnosis and management of GDM maternal- and neonatal-associated complications, considerably contributing to health care costs in both developed and developing economies [3, 4]. Globally, the 2005–2015 prevalence of GDM varied by different regions, with the Middle East, North Africa, Southeast Asia and the Western Pacific having the greatest median prevalence of 12–13% and Europe having the lowest median prevalence of 6% [5]. In multi-cultural Australia, GDM rates trebled over the past two decades rising from 5% in 2000 to 15% in 2017 [6]. Increasing trends in prevalence of GDM have also been reported in Britain [7], Europe [8], North America [9], and China [10].

The worldwide increase in the prevalence of GDM can be partially explained by increased obesity rates and rising maternal age among childbearing women observed in high- and middle-income countries [11–13]. In countries such as the United Kingdom, the United States and Australia, approximately 50% of childbearing women live with overweight or obesity [14], with Australia having the 11th highest proportion of overweight or obesity in women among OECD member countries in 2017 [15]. An Australian study, conducted in metropolitan Sydney, reported an increase over time in the percentages of gestational diabetes attributable to overweight and obesity rising from 12.9% in 1990–1994 to 17.0% in 2010–2014 [16]. However, in parts of regional and rural Australia where overweight, obesity and morbid obesity are more prevalent [17], compounded with socioeconomic disadvantage and higher migrant communities, trends over time in adjusted percentages of GDM attributed to obesity and morbid obesity are not known.

Using routinely collected hospital data, this 8-year retrospective panel study explored secular trends in GDM attributable to overweight, obesity, and morbid obesity in a large population-based sample in regional Victoria, Australia. Secular trends in GDM were evaluated by age and body mass index categories. The characteristics of expecting mothers over time were explored and risk factors associated with GDM were examined.

## Methods

### Study sample

The study sample and setting have been described previously [18]. Briefly, all women birthing at a large tertiary hospital in regional Victoria, Australia between January 2010 and December 2017 were eligible for inclusion. Women with type 1 or 2 diabetes were excluded. Women with multiple pregnancies, or pregnancies that resulted in singletons but that started as multiple, were also excluded. Women with missing weight or height were excluded. All study variables were extracted from the Birthing Outcome System (BOS) database, an electronic, hospital-based integrated data collection tool that facilitates longitudinal patient data recording on socio-demographics, antenatal, intrapartum, and postpartum information relating to each birth [12]. Missing information was not common as all study variables were entered into compulsory data fields that were completed by clinicians and administrative staff.

### Definitions and study variables

Each woman was followed from her first antenatal visit until her hospital discharge following birth. Women with GDM were identified through an algorithm which utilised information from free-text terms recorded in at least one of three fields in BOS: obstetric complication, labour complication, and reason for induction. The free-text strings used to identify the women were: gestational diabetes, gestational diabetes mellitus, gestational DM, and GDM. Identification of diagnoses using free-text algorithms is widely used in Australia in studies that utilise routinely collected medical and administrative data [12, 19, 20].

Study variables included age, body mass index (BMI), country of birth, Socio-Economic Indexes for Areas – Index of Relative Socio-Economic Disadvantage (SEIFA-IRSD), Indigenous status (as self-identified as Aboriginal or Torres Strait Islander), year of delivery, smoking, parity, gravidity, hypertension, polycystic ovary syndrome, gestational hypertension, gestational diabetes, pre-eclampsia or eclampsia, number of ultrasounds conducted during pregnancy, and past history of GDM for multiparous women. Age was summarised into fifths based on its distribution in the sample. BMI was estimated using the weight and height measured by the midwife at the woman’s first antenatal visit. Socioeconomic status was defined by SEIFA-IRSD obtained from the Australian 2006 and 2011 Census data. SEIFA-IRSD is a composite index of relative advantage or disadvantage based on geographic areas across Australia, with higher scores indicating less socio-economic disadvantage [21]. The index was grouped into its fifths according to the score’s distribution in the sample.

The diagnostic criteria of gestational diabetes in Australia changed during the study period. In the hospital where the data of this study come from, during 2010–2015, the diagnosis was based on the Australasian Diabetes in Pregnancy Society (ADIPS) criteria [22]; 2016 onwards, the International Association of the Diabetes in Pregnancy Study Groups (IADPSG) criteria were endorsed [23]. The diagnosis of GDM was made by clinicians at the hospital following the recommended criteria for each year. The diagnostic criteria for both time periods are shown in Supplementary Table 1. GDM cases diagnosed within and beyond the 24–28 gestation period were captured.

### Statistical analyses

Occurrence of GDM (yes/no) was measured for each woman in each year, with adjusted percentages, derived from a logistic regression applied on the whole sample, plotted over time by BMI and age categories. Using an exchangeable working covariance matrix, GDM was modelled using a generalised estimating equations (GEE) logistic regression which accounted for correlation and dependence between repeat deliveries on the same individual over time while adjusting for study variables associated with the study outcome in univariate tests with a *p* value < 0.1.

The percentages of GDM that could have been avoided with the elimination of maternal overweight, obesity, and morbid obesity were estimated using model-based population attributable fractions (AFp) for the whole sample and by year of delivery. The AFp together with its confidence intervals were estimated from the ratio of the logs of conditional means of possible scenarios as recommended by Greenland and Drescher [24]. Change in GDM incidence over time and change in adjusted AFp associated with overweight, obesity, and morbid obesity were tested using the augmented Dickey-Fuller test [25]. To fail to reject the null hypothesis and conclude that time series is non-stationary, the Mackinnon approximate *p* value of this test needs to be insignificant (i.e., larger than 0.05) [25].

The dose–response effects of BMI categories on GDM were tested for the first delivery in the 8-year study period using likelihood ratio tests, with nested regression models being compared to capture data trends. An insignificant *p* value of the log likelihood test indicates linearity [26].

### Scenario analysis

To account for the change in the GDM diagnostic criteria during the study period, we conducted a scenario analysis in which we predicted the GDM status using simulations. Two separate simulations were conducted:

1. GDM diagnosis in 2016 and 2017 was made missing. Using the GDM status in years 2010–2015 (i.e., based on ADIPS criteria), we predicted the GDM status of women birthing in years 2016 and 2017.
2. GDM diagnosis in years 2010–2015 was made missing. Using the GDM status in years 2016–2017 (i.e., based on IADPSG/WHO diagnostic criteria), we predicted the GDM status of women birthing in years 2010–2015.

We used chained equations utilising all study known variables to generate the GDM status (a yes/no variable), with 50 generated datasets and final estimates obtained using Rubin’s rules [27], which accounted for the variability in the predicted values among the generated datasets. To avoid bias in generating the predicted values [28], all study variables including year of delivery were included in the prediction model. Women’s characteristics which changed over time were accounted for in the models. Following each of the simulations, we estimated and plotted the percentages of GDM attributable to obesity over the years as conducted in epidemiological studies [29].

The analyses were performed using Stata/SE 16 (Stata Corp LP., College Station TX, USA).

### Ethics clearance

Ethics clearance was obtained from Bendigo Health Human Research Ethics Committee (reference number LNR/16/BHCG/50) in April 2017 with amendments accepted in July 2020. Informed consent was waived by Bendigo Health Human Research Ethics Committee and La Trobe University Human Research Ethics Committee.

## Results

During the 8-year study period, a total of 7495 women experienced a singleton birth, of whom 69.0% gave birth once. Of the 7495 women, 81 (1.1%) with pre-existing diabetes and 66 (0.9%) with a missing weight or height were excluded, leaving a sample of 7348 women, contributing to 10,028 births, for analysis.

Baseline (i.e., at first delivery in study period) characteristics of the sample by BMI categories are presented in Table 1. Compared to women with BMI ≤25 kg/m^2^, women with obesity and morbid obesity were older, came from more disadvantaged socioeconomic backgrounds, had more comorbidities, and underwent more ultrasound tests during their pregnancies. Approximately 32% of women born in Australia, New Zealand, Europe, or the Americas had a BMI ≥ 30.0 kg/m^2^. Women born in East Asia, South Asia, or Southeast Asia were the leanest, with obesity prevalence of 7.9%.

**Table 1:** Characteristics of women at first delivery by body mass index (BMI) category

	BMI < 25 kg/m^**2**^ ***N*** = 3159 (43.0%)	BMI 25.0–29.9 kg/m^**2**^ ***N*** = 2036 (27.7%)	BMI 30.0–34.9 kg/m^**2**^ ***N*** = 1178 (16.0%)	BMI ≥ 35 kg/m^**2**^ ***N*** = 975 (13.3%)	P value
**Age categories**, years					< 0.001
≤ 24 (youngest: 14 years)	897 (28.4)	428 (21.0)	297 (25.2)	212 (21.7)	
25–27	536 (17.0)	347 (17.0)	204 (17.3)	167 (17.1)	
28–30	626 (19.8)	401 (19.7)	224 (19.0)	181 (18.6)	
31–34	605 (19.2)	448 (22.0)	212 (18.0)	194 (19.9)	
≥ 35 (oldest: 50 years)	495 (15.7)	412 (20.2)	241 (20.5)	221 (22.7)	
**Country of birth**					< 0.001
Australia – non-Indigenous	2563 (81.1)	1762 (86.5)	1043 (88.5)	866 (88.8)	
Australia – Indigenous	183 (5.8)	90 (4.4)	64 (5.4)	71 (7.3)	
East/Southeast Asia	177 (5.6)	56 (2.8)	11 (0.9)	3 (0.3)	
South Asia	111 (3.5)	49 (2.4)	16 (1.4)	4 (0.4)	
Europe / Americas	79 (2.5)	33 (1.6)	18 (1.5)	11 (1.1)	
Polynesia	17 (0.5)	15 (0.7)	10 (0.9)	13 (1.3)	
Middle East / Africa	19 (0.6)	20 (1.0)	11 (0.9)	3 (0.3)	
Unknown	10 (0.3)	11 (0.5)	5 (0.4)	4 (0.4)	
**Socioeconomic status**					< 0.001
High	927 (29.3)	692 (34.0)	350 (29.7)	275 (28.2)	
Middle	1200 (38.0)	659 (32.4)	399 (33.9)	334 (34.3)	
Low	1032 (32.7)	685 (33.6)	429 (36.4)	366 (37.5)	
**Past or present smoker**	702 (22.2)	408 (20.0)	277 (23.5)	210 (21.5)	0.107
**Primiparous**	1845 (58.4)	1080 (53.1)	616 (52.3)	443 (45.4)	< 0.001
**Gravida categories**					< 0.001
1	1421 (45.0)	800 (39.3)	456 (38.7)	327 (33.5)	
2	815 (25.8)	510 (25.1)	286 (24.3)	245 (25.1)	
≥ 3	923 (29.2)	726 (35.7)	436 (37.0)	403 (41.3)	
**Pre-existing hypertension**	25 (0.8)	15 (0.7)	25 (2.1)	47 (4.8)	< 0.001
**Polycystic ovary syndrome**	73 (2.3)	73 (3.6)	75 (6.4)	108 (11.1)	< 0.001
**Number of ultrasound tests during pregnancy**					< 0.001
0	160 (5.1)	85 (4.2)	45 (3.8)	39 (4.0)	
1	454 (14.4)	279 (13.7)	175 (14.9)	131 (13.4)	
2	994 (31.5)	628 (30.8)	350 (29.7)	262 (26.9)	
3	890 (28.2)	604 (29.7)	327 (27.8)	263 (27.0)	
≥ 4	661 (20.9)	440 (21.6)	281 (23.9)	280 (28.7)	

The proportion of older women, and women with obesity increased over time. In 2010, 42.9% were aged ≥30 years, increasing to 49.9% in 2017, *p* <  0.001; whereas 29.0% had a BMI ≥ 30.0 kg/m^2^ in 2010 increasing by 11 to 32.2%, in 2017 *p* = 0.023. Women born overseas and women born in South Asia, Southeast Asia and East Asia increased over time (*p* <  0.001). Induction of labour, emergency caesarean sections also increased over time (*p* <  0.001 in both) (Table 2).

**Table 2:** Women’s characteristics over time: *N* = 10,028 live singleton births

	All N = 10,028	2010 ***N*** = 1117	2011 ***N*** = 1171	2012 ***N*** = 1168	2013 ***N*** = 1276	2014 ***N*** = 1299	2015 ***N*** = 1255	2016 ***N*** = 1321	2017 ***N*** = 1421	p
**Age ≥ 30**, %	46.0	42.9	43.0	43.1	45.1	46.3	47.7	48.9	49.9	< 0.001
**BMI ≥ 30.0 kg/m**^**2**^, %	30.2	29.0	26.1	29.0	31.9	31.2	30.0	31.2	32.2	0.023
**Born overseas**, %	8.2	6.2	7.9	6.3	9.0	7.5	9.6	9.2	9.6	0.002
**Born in South Asia / East Asia / Southeast Asia**, %	5.5	3.0	3.4	4.8	5.7	5.1	7.4	6.7	7.0	< 0.001
**Pre-existing hypertension**, %	1.5	2.1	1.5	1.5	1.6	1.4	1.4	1.6	0.9	0.523
**Number of ultrasounds during pregnancy ≥ 4**, %	22.6	22.6	14.3	10.9	18.0	17.6	20.2	32.9	40.5	< 0.001
**GDM by BMI category (kg/m**^**2**^**)**, %										< 0.001
< 25.0	4.7	1.4	1.4	2.6	5.2	5.1	8.5	6.1	7.0	
25.0–29.9	7.5	3.4	1.3	5.0	9.7	6.5	11.1	11.1	9.6	
30.0–34.9	13.8	4.7	4.9	7.8	14.9	13.2	16.7	23.7	20.6	
≥ 35.0	21.6	9.1	5.8	16.5	18.6	24.2	29.8	26.9	30.8	
**GDM management with oral hypoglycaemic agents**, %	1.8	0.0	0.1	0.7	1.8	1.6	3.0	3.8	3.1	< 0.001
**GDM management with insulin**, %	2.5	1.1	0.7	1.9	2.7	2.2	3.9	4.2	3.2	< 0.001
**Induction of labour**, %	27.8	23.3	23.0	24.1	26.5	29.0	29.0	31.0	34.7	< 0.001
**Emergency caesarean**, %	14.9	11.6	12.0	14.4	15.1	15.4	15.1	18.2	16.3	< 0.001

*Abbreviations*: *BMI* body mass index, *GDM* gestational diabetes mellitus

**Fig. 1:**
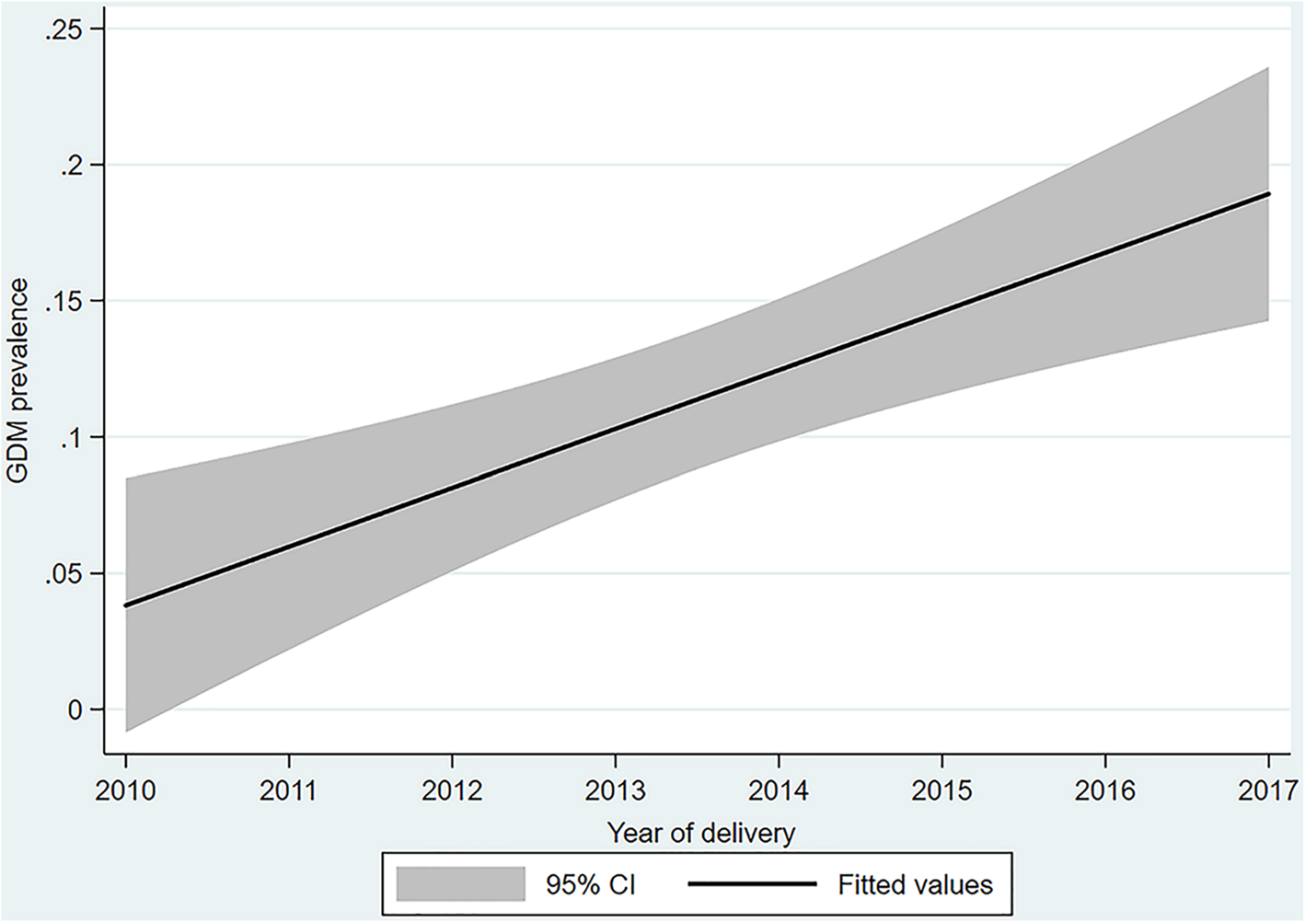
Incidence of GDM over time, all deliveries during the eight-year period

**Fig. 2:**
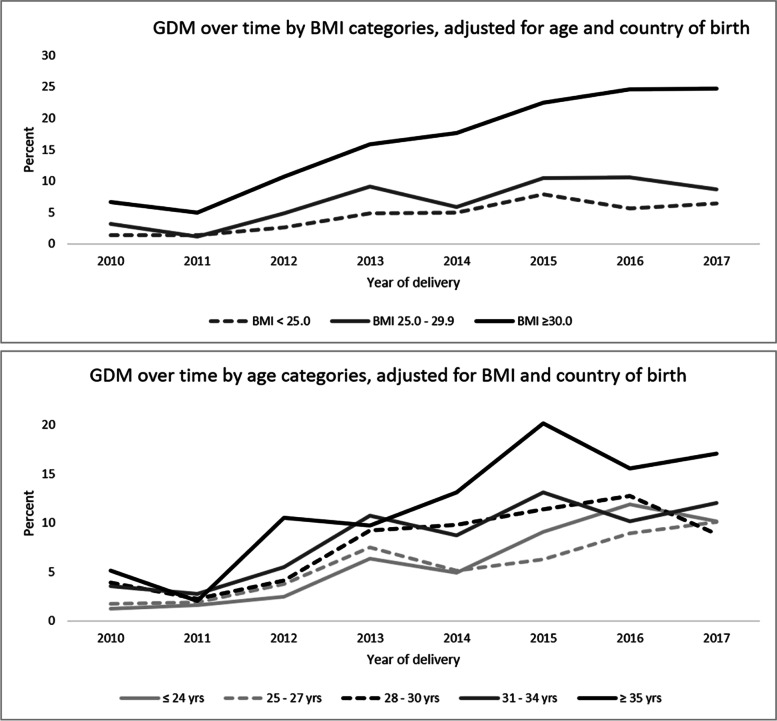
GDM percentages over time^!^ by body mass index and age categories.^!^ Increased trends over time were observed in all except the age group ≤24 years

Of the 10,028 births, GDM was diagnosed among 930 (9.3%), significantly increasing over time (Fig. 1). Increased incidence was evident in different BMI and age categories (Fig. 2) with increasing trends found in each BMI category and in all ages except women aged ≤24 years.

**Fig. 3:**
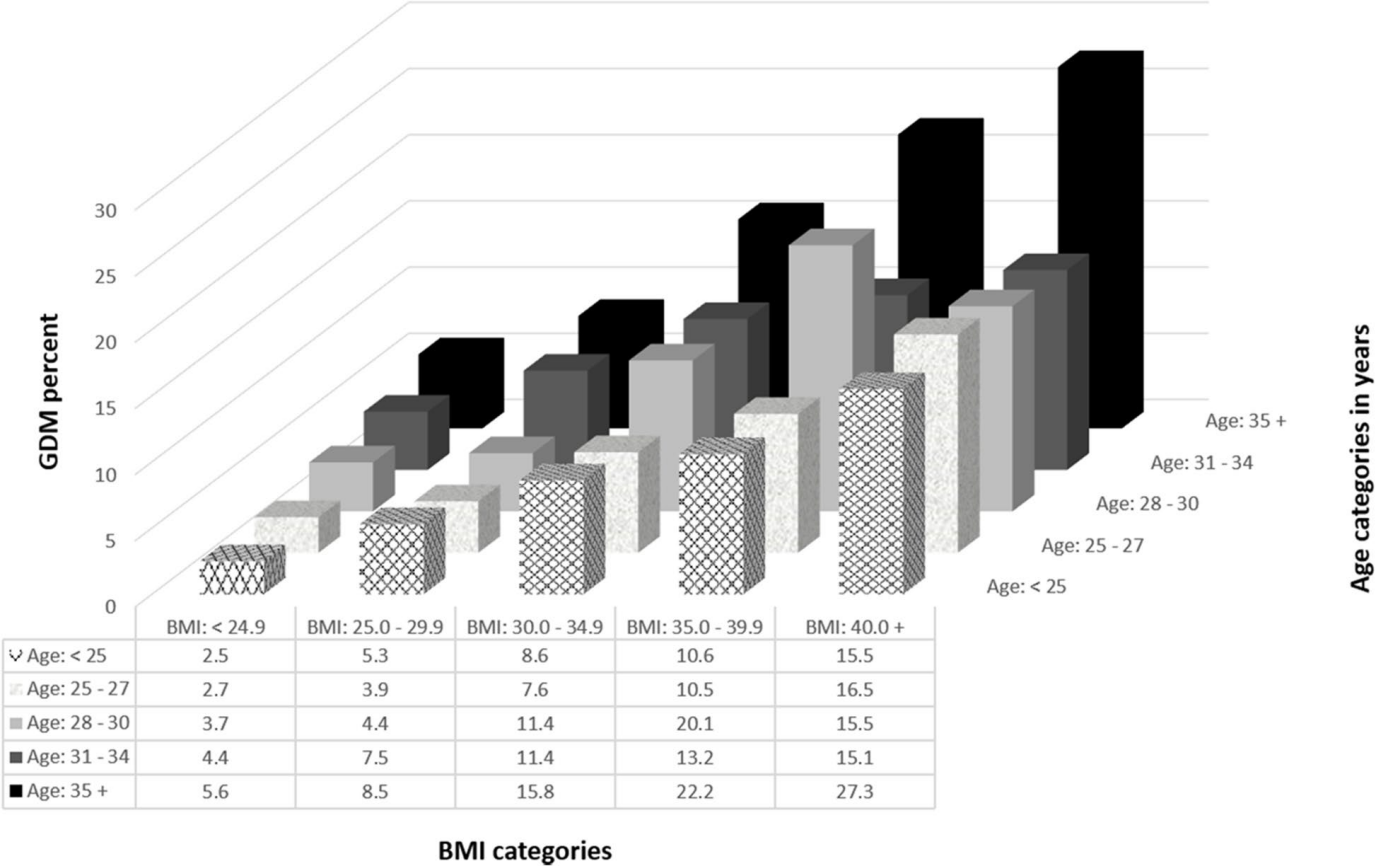
GDM percentages^!^ by combinations of age and BMI categories.^!^ The percentages were adjusted for country of birth, socioeconomic status, Indigenous status, smoking, pre-existing hypertension, past history of gestational diabetes, parity, gravidity, polycystic ovary syndrome, and number of ultrasounds during pregnancy

Percentages of GDM by different combinations of age and BMI categories are shown in Fig. 3, with highest expected percentages found among women with morbid obesity who were ≥ 35 years old (27.3% compared to 2.5% among women aged < 25 years with BMI < 24.9, (*p* <  0.001).

In the multivariable analysis, odds of GDM increased in a dose-response manner with increasing BMI (Table 3).

**Table 3:** Odds ratios for having gestational diabetes at any time in the 8 years (including first and repeated births per woman)

	N of births	Unadjusted analyses		Multivariable analyses^**a**^	
Variable	N (%)	OR (95% CI)	***P***	OR (95% CI)	***P***
**Age categories (**fifths, based on age distribution in sample), years					
1st fifth: < 25 (youngest: 14 years)	2301 (23.0)	1.00		1.00	
2nd fifth: 25–27	1740 (17.4)	1.14 (0.90–1.45)	0.273	0.92 (0.70–1.19)	0.515
3rd fifth: 28–30	2056 (20.5)	1.50 (1.19–1.87)	< 0.001	1.27 (0.99–1.62)	0.060
4th fifth: 31–34	2115 (21.1)	1.69 (1.36–2.11)	< 0.001	1.31 (1.02–1.69)	0.033
5th fifth: ≥35 (oldest: 50 years)	1816 (18.1)	2.60 (2.09–3.23)	< 0.001	1.96 (1.53–2.51)	< 0.001
**BMI**, kg/m^2^					
< 25.0	4198 (41.9)	1.00		1.00	
25.0–29.9	2804 (28.0)	1.67 (1.36–2.04)	< 0.001	1.68 (1.36–2.07)	< 0.001
30.0–34.9	1671 (16.7)	3.26 (2.66–4.00)	< 0.001	3.43 (2.77–4.25)	< 0.001
≥35	1355 (13.5)	5.51 (4.51–6.74)	< 0.001	5.53 (4.47–6.84)	< 0.001
**Country of birth**					
Australia	9122 (91.0)	1.00		1.00	
East/Southeast Asia	326 (3.3)	1.50 (1.06–2.13)	0.023	2.12 (1.41–3.19)	< 0.001
South Asia Europe / Americas Polynesia Middle East / Africa Unknown	216 (2.2) 183 (1.8) 74 (0.7) 69 (0.7) 38 (0.4)	3.90 (2.83–5.39) 1.43 (0.87–2.34) 2.52 (1.36–4.70) 1.81 (0.88–3.72) 0.99 (0.30–3.28)	< 0.001 0.161 0.004 0.108 0.988	5.71 (3.97–8.20) 2.00 (1.18–3.37) 3.21 (1.71–6.04) 2.54 (1.22–5.30) 1.11 (0.32–3.85)	< 0.001 0.010 < 0.001 0.013 0.865
**Year of delivery**					
2010	1117 (11.1)	1.00		1.00	
2011	1171 (11.7)	0.70 (0.44–1.12)	0.134	0.70 (0.42–1.18)	0.180
2012	1168 (11.7)	1.69 (1.17–2.44)	0.005	1.84 (1.23–2.76)	0.003
2013	1276 (12.7)	3.11 (2.22–4.38)	< 0.001	2.95 (2.04–4.27)	< 0.001
2014	1299 (13.0)	2.97 (2.11–4.19)	< 0.001	2.64 (1.82–3.83)	< 0.001
2015	1255 (12.5)	4.39 (3.13–6.14)	< 0.001	3.88 (2.70–5.59)	< 0.001
2016	1321 (13.2)	4.25 (3.03–5.96)	< 0.001	3.25 (2.25–4.71)	< 0.001
2017	1421 (14.2)	4.42 (3.16–6.17)	< 0.001	3.06 (2.12–4.41)	< 0.001

^**a**^ Generalised estimating equations (GEE) logistic regression, also adjusting for socioeconomic status, Indigenous status, smoking, pre-existing hypertension, past history of gestational diabetes, parity, gravidity, polycystic ovary syndrome, and number of ultrasounds during pregnancy

Population attributable fraction analyses estimated that 8.6% (confidence interval (CI) 6.1–11.0%), 15.6% (95% CI 12.2–19.0%), and 19.5% (95% CI 15.3–23.6%) of GDM would have been prevented by eliminating maternal overweight, obesity, and morbid obesity, respectively. The unadjusted and risk-adjusted population attributable fractions are shown in Table 4.

**Table 4:** Unadjusted and risk-adjusted population attributable fractions by different comparator scenarios

Comparators	Unadjusted AFp (95% CI)	Risk-adjusted AFp (95% CI)
**Overweight versus normal weight**	9.3% (6.7–11.9%)	8.6% (6.1–11.0%)
**Obesity versus normal weight**	17.1% (13.3–20.8%)	15.6% (12.2–19.0%)
**Morbid obesity versus normal weight**	21.3% (16.7–25.7%)	19.5% (15.3–23.6%)

*Abbreviation*: *AFp* Population Attributable Fraction

**Fig. 4:**
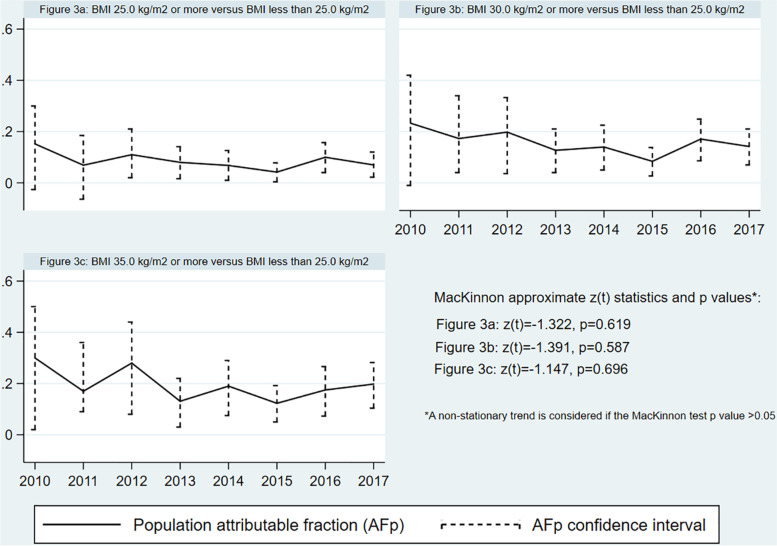
Percentages of gestational diabetes mellitus attributable to overweight, obesity, and morbid obesity over time

Despite the significant increase in obesity over time, burden of GDM associated with overweight, obesity, or morbid obesity significantly dropped over time as supported by the MacKinnon approximate tests shown in Fig. 4. In 2010, the percentages of GDM attributable to obesity and morbid obesity were respectively 23.3% (CI -1.0-42.0%) and 30% (CI 2.0–5.0%) dropping to 14.2% (CI 7.0–21.0%) and 19.8% (CI 10.4–28.2%) in 2017.

The scenario analyses supported the decreasing trends in percentages of GDM attributed to obesity as shown in Supplementary Fig. 1.

## Discussion

In a population-based longitudinal study of 10,028 mother-child pairs utilising routinely collected hospital data, this study provides evidence of a significant increase in GDM trends detected over a relatively short period of 8 years. Although GDM incidence was consistently highest among women with obesity and morbid obesity and although obesity and GDM significantly increased over time, burden of disease associated with obesity or morbid obesity dropped over time. These findings may indicate an increase over time in risk factors for GDM other than obesity.

Similar to other studies [30, 31], using a large Australian sample we report increasing secular trends in the incidence of gestational diabetes mellitus with some of this increase attributed to increasing maternal obesity and to older maternal age at delivery. The increasing trends were also demonstrated after adjusting for sociodemographic, past obstetric history, and pre-existing comorbidities. Our found independent associations between higher BMI, older maternal age, and increased risk of GDM are well documented [32], with risk of GDM being nearly 11 times higher among women with morbid obesity (BMI > 40 kg/m^2^) aged ≥35 years than that in leaner women (BMI < 25 kg/m^2^) aged < 25 years. Our estimated percentage of GDM attributable to obesity is similar to another Australian study [16] but is considerably lower than the 46% reported by Kim et al. [13] although our GDM rates are much higher than those reported by these authors. The differences between our and Kim et al. findings could have resulted from the much higher GDM rates in women with normal BMI (the reference group) in our sample. However, similar to these authors, our study confirms the increased risk of GDM associated with increasing BMI. In our data, obesity or morbid obesity was more common amongst women born in Australia and other western economies. Women born in East Asia, South Asia, or Southeast Asia were the leanest. Nonetheless, risk of GDM was significantly and independently higher among these migrant women. Although this study adjusted for country of birth, besides the Indigenous status, the ethnicities of the women were unknown to us. Ethnicity may have been one of the major factors contributing to the increase in the incidence of GDM over time. Just over half of the Indigenous Victorians reside in regional and rural locations and there are increasing numbers of ethnic minorities including migrants, refugees, and Australian-born non-Caucasian ethnic communities settling in regional Victoria with these communities contributing to population growth in regional Australia [33]. Our findings suggest that ethnicity, acculturation together with changes in lifestyle and environmental factors may have increased the risk of GDM among migrant populations and ethnic minorities [34–36]. Although the reasons underlying the ethnic differences remain unclear, genetic factors and glucose metabolism have been suggested as a possible explanation [37].

Our large-scale population-based study found no evidence to indicate that the new diagnostic criteria (IADPSG) had had any impact on the detection of GDM. The overall GDM incidence remained constant the year before, during the same year, and in the year after the new criteria were implemented, being 13.6% in 2015, 13.5% in 2016 (the year the new criteria were adopted), and 13.6% in 2017, *p* = 0.991. Although some medical centres in Victoria, Australia, reported an increase in GDM diagnosis after the new criteria were used [38], no differences were found in our study. A multi-centre study (the Hyperglycaemia and Adverse Pregnancy Outcome (HAPO) study) that examined the frequency of GDM in 15 medical centres in nine countries using the new IADPSG criteria found a wide centre-to-centre variation (9.3 to 25.5%) in the proportion of pregnant women diagnosed with GDM – a variation that persisted after adjusting for maternal age, BMI, family history of diabetes mellitus, and hypertension [39]. The authors could not explain the variation in GDM diagnosis across the centres suggesting that factors relating to glucose metabolism could have contributed to these differences. Differences in testing among the medical centres may also contribute to this variation with evidence indicating that centres across Australia increased universal testing following the implementation of the new diagnostic criteria [40]. Since their recommendation, these criteria have been tested and validated in different populations worldwide including countries with predominantly Asian populations [41–44]. However, when these criteria were compared against pregnancy or neonatal adverse outcomes, the validation results were inconsistent. Some studies found significant associations between the criteria and adverse neonatal outcomes [41, 42], while others failed to detect an association [43, 44]. It was suggested that these diagnostic criteria might miss a proportion of cases with abnormal glucose metabolism that could lead to increased neonatal adverse outcomes due to untreated GDM [45].

### Strengths and limitations

Strengths of this study include its population-based provenance, the generalisability of the results, longitudinal design, and the usage of readily available routinely collected hospital data. However, the study also has limitations. As earlier stated, the ethnicities of the women were unknown to us. Maternal BMI was measured at the first antenatal visit; we had no information on weight gain during the pregnancy; however, BMI at the first antenatal visit has been shown to be a valid measure to predict gestational diabetes [16]. Similarly, we had no information on the gestational age of women at the first antenatal booking. We did not have access to pathology results and the diagnosis of GDM solely relied on the face validity of diagnoses made by obstetricians and gynaecologists at the hospital that were recorded in the hospital electronic files.

## Conclusions

Describing population trends of GDM using hospital data offers advantages in regulatory surveillance of GDM at a population level which can assist to improve health planning and explore prevention strategies. This study provides evidence of increasing GDM proportions over time together with a change in the characteristics of expecting mothers over time is a large representative Australian regional population. Although GDM attributable to obesity was clearly demonstrated in our study, the percentage of GDM attributable to obesity dropped over time despite the significant increase in obesity over time. This may indicate that other-than-obesity risk factors for GDM may be increasing over time. Accounting for change in the case-mix is critical to better predict GDM and to improve medical care [46] while controlling for varying characteristics over time. GDM is a multifactorial disease; better knowledge of the risk profile could optimise the early and adequate management of women at higher risk for GDM.

## Supplementary Information

**Additional file 1.**

## Data Availability

All data generated or analysed during this study are included in this published article (and its supplementary information files).

## Abbreviations

ADIPS: Australasian Diabetes in Pregnancy Society
AFp: Population attributable fractions
BMI: Body mass index
BOS: Birthing Outcome System
GDM: Gestational diabetes mellitus
GEE: Generalised estimating equations
IDF: International Diabetes Federation
SEIFA-IRSD: Socio-Economic Indexes for Areas – Index of Relative Socio-Economic Disadvantage
