# Supplementary material for: Trends in percentages of gestational diabetes mellitus attributable to overweight, obesity, and morbid obesity in regional Victoria: an eight-year population-based panel study
Source: BMC Pregnancy Childbirth. 2022 Feb 1;22:95. doi: 10.1186/s12884-022-04420-9 (PMC8809044; doi:10.1186/s12884-022-04420-9)

**Trends in percentages of gestational diabetes mellitus attributable to overweight, obesity, and morbid obesity in regional Victoria: an eight-year population-based panel study**

George Mnatzaganian, Mark Woodward, H David McIntyre, Liangkun Ma, Nicola Yuen, Fan He, Helen Nightingale, Tingting Xu, Rachel R Huxley

**Supplementary Table 1**: Diagnostic criteria for gestational diabetes mellitus in Australia

| Criteria | Fasting glucose (mmol/L) | 1-hour^!^ glucose (mmol/L) | 2-hour^!^ glucose (mmol/L) |
| --- | --- | --- | --- |
| ADIPS^21^ | ≥5.5 | N/A | ≥8.0 |
| IADPSG/WHO^22 $^ | ≥5.1 | ≥10.0 | ≥8.5 |

Abbreviations: ADIPS, Australasian Diabetes in Pregnancy Society; IADPSG, International Association of the Diabetes in Pregnancy Study Groups; N/A, not applicable, WHO, World Health Organization.

! Glucose levels following 75 g glucose tolerance test

$ The IADPSG/WHO criteria were endorsed by the study participating hospital in 2016.

**Supplementary Figure 1**: Percentages of gestational diabetes mellitus attributable to obesity: scenario analysis


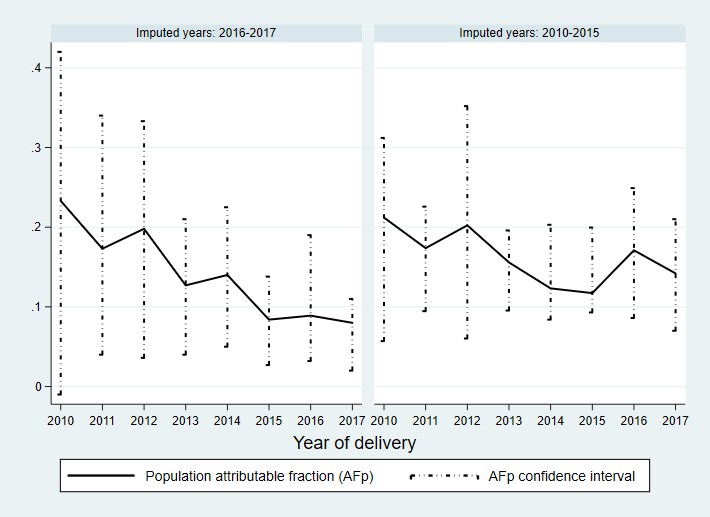

Supplement: Supplementary file 1 — Additional file 1. [file 12884_2022_4420_MOESM1_ESM.docx]
